# Social, economic, and health effects of the COVID-19 pandemic on adolescents retained in or recently disengaged from HIV care in Kenya

**DOI:** 10.1371/journal.pone.0257210

**Published:** 2021-09-10

**Authors:** Leslie A. Enane, Edith Apondi, Josephine Aluoch, Giorgos Bakoyannis, Jayne Lewis Kulzer, Zachary Kwena, Rami Kantor, Ashley Chory, Adrian Gardner, Michael Scanlon, Suzanne Goodrich, Kara Wools-Kaloustian, Batya Elul, Rachel C. Vreeman

**Affiliations:** 1 Department of Pediatrics, The Ryan White Center for Pediatric Infectious Disease and Global Health, Indiana University School of Medicine, Indianapolis, Indiana, United States of America; 2 Indiana University Center for Global Health, Indianapolis, Indiana, United States of America; 3 Academic Model Providing Access to Healthcare (AMPATH), Eldoret, Kenya; 4 Moi Teaching and Referral Hospital, Eldoret, Kenya; 5 Department of Biostatistics and Health Data Science, Indiana University, Indianapolis, Indiana, United States of America; 6 Department of Obstetrics, Gynecology, and Reproductive Sciences, University of California San Francisco, San Francisco, California, United States of America; 7 Research, Care and Treatment Programme, Centre for Microbiology Research, Kenya Medical Research Institute, Nairobi, Kenya; 8 Division of Infectious Diseases, Department of Medicine, Brown University Apert Medical School, Providence, Rhode Island, United States of America; 9 Department of Health System Design and Global Health, Icahn School of Medicine at Mount Sinai, New York, New York, United States of America; 10 Arnhold Institute for Global Health, New York, New York, United States of America; 11 Division of Infectious Diseases, Department of Medicine, Indiana University School of Medicine, Indianapolis, Indiana, United States of America; 12 Department of Epidemiology, Mailman School of Public Health, Columbia University, New York, New York, United States of America; Emory University School of Medicine, UNITED STATES

## Abstract

**Introduction:**

Adolescents living with HIV (ALHIV, ages 10–19) experience complex challenges to adhere to antiretroviral therapy (ART) and remain in care, and may be vulnerable to wide-scale disruptions during the COVID-19 pandemic. We assessed for a range of effects of the pandemic on ALHIV in western Kenya, and whether effects were greater for ALHIV with recent histories of being lost to program (LTP).

**Methods:**

ALHIV were recruited from an ongoing prospective study at 3 sites in western Kenya. The parent study enrolled participants from February 2019–September 2020, into groups of ALHIV either 1) retained in care or 2) LTP and traced in the community. Phone interviews from July 2020–January 2021 assessed effects of the pandemic on financial and food security, healthcare access and behaviors, and mental health. Responses were compared among the parent study groups.

**Results:**

Phone surveys were completed with 334 ALHIV or their caregivers, including 275/308 (89.3%) in the retained group and 59/70 (84.3%) among those LTP at initial enrollment. During the pandemic, a greater proportion of LTP adolescents were no longer engaged in school (45.8% vs. 36.4%, p = 0.017). Over a third (120, 35.9%) of adolescents reported lost income for someone they relied on. In total, 135 (40.4%) did not have enough food either some (121, 36.2%) or most (14, 4.2%) of the time. More LTP adolescents (4/59, 6.8% vs. 2/275, 0.7%, p = 0.010) reported increased difficulties refilling ART. Adolescent PHQ-2 and GAD-2 scores were ≥3 for 5.6% and 5.2%, respectively.

**Conclusions:**

The COVID-19 pandemic has had devastating socioeconomic effects for Kenyan ALHIV and their households. ALHIV with recent care disengagement may be especially vulnerable. Meanwhile, sustained ART access and adherence potentially signal resilience and strengths of ALHIV and their care programs. Findings from this survey indicate the critical need for support to ALHIV during this crisis.

## Introduction

The COVID-19 pandemic has led to massive disruptions to economies, health systems, and social life across the globe. Emerging evidence indicates detrimental effects of the COVID-19 pandemic on health funding, systems, and access, which may exacerbate vulnerabilities among children and youth in sub-Saharan Africa [1–7]. Adolescents living with HIV (ALHIV, ages 10–19), who are already at higher risk of poor health outcomes relative to younger and older people living with HIV (PLHIV), are strongly influenced by structural barriers to care and social determinants of health [8–11]. Complex adolescent-specific challenges to adhering to antiretroviral therapy (ART) and remaining in HIV care [9, 11] may therefore be compounded during the pandemic [4, 12–17].

Given the significant vulnerabilities and specific healthcare needs of ALHIV, the COVID-19 pandemic could have significant effects on adolescent well-being and HIV outcomes [18, 19]. Further, adolescents are at high risk for mental health challenges related to HIV stigma, isolation, and trauma [8, 20–22], and these may be heightened during disruptions to schooling, work, and social activities during the pandemic. Economic downturns resulting from the pandemic may leave ALHIV and their families vulnerable to food or housing insecurity, which can further complicate engagement in HIV care. Such consequences, seen in prior crises, must be characterized and addressed in the current pandemic, in preparation for future, inevitable occurrences [23].

Kenya reported its first case of COVID-19 on March 13, 2020 [24], and has experienced three “waves” of increased COVID-19 incidence: in July/August, 2020; October/November 2020; and as of this writing a third wave was occurring in March/April 2021 [25, 26]. Kenya implemented several mitigation strategies in March 2020, including border closures; a ban on international travel, social gatherings, closures of schools, religious places, bars, and restaurants; movement restrictions; and a curfew [27]. Some restrictions were lifted in June 2020; however, the most recent wave of the pandemic has prompted reinstatement of movement restrictions, closures, and a curfew [27, 28]. A prior large-scale crisis in Kenya—the 2007–2008 post-election crisis—prompted similar restrictions and resulted in challenges to retention in HIV care and ART adherence among PLHIV, impacting viral suppression and drug resistance [23, 29–33]. The effect of the ongoing COVID-19 pandemic on PLHIV in Kenya is not yet fully known.

Amid increasing COVID-19 case numbers in Kenya [25–27], we assessed effects of the pandemic on the social, economic, HIV care, physical, and mental wellbeing of ALHIV who were either retained in care or lost to program (LTP) at enrollment in an ongoing prospective study. We hypothesized that ALHIV may experience significant psychosocial effects of the pandemic, and that ALHIV with histories of care disengagement may be particularly vulnerable amid health system, economic, and social disruptions.

## Methods

### Setting and study population

We implemented a phone survey among ALHIV participating in a cohort study in western Kenya [34]. The parent study intentionally enrolled two ALHIV cohorts: those engaged in HIV care at enrollment and those who had been engaged in HIV care within the year but were LTP at the time of enrollment. That study aims to assess drivers of HIV care disengagement among ALHIV. This ongoing study is being conducted at two sites in the Academic Model Providing Access to Healthcare (AMPATH), in Eldoret and Kitale, and also at FACES Lumumba in Kisumu, through the NIH-funded East Africa International Epidemiologic Databases to Evaluate AIDS (IeDEA) Regional Consortium (U01 AI069911).

Enrollment for the parent study occurred from February 2019 to September 2020. Inclusion criteria were: ages 10–19; enrolled in care at one of the study sites; and attendance at ≥1 visit in the 18 months prior to study enrollment. The LTP group was sought from a comprehensive sample of ALHIV who had not attended clinic ≥60 days following a missed scheduled visit. Those ALHIV and their caregivers were traced by community health volunteers and study staff through phone calls and home visits. Adolescents identified as disengaged from care during tracing were encouraged and facilitated to re-engage in care. The retained ALHIV were recruited from a random sample of eligible ALHIV at the study sites.

### Approach to phone surveys

We developed a phone survey to assess a range of potential effects, including impacts on financial and food security, healthcare access, health behaviors, adherence to ART, and mental health. The survey was designed to be as brief as possible for feasibility of conducting surveys by phone. Survey questions were largely closed-ended, but given that the study was designed and implemented as the COVID-19 pandemic was rapidly evolving, we also included open-ended questions to capture acute and emerging challenges which might not have been anticipated when the questionnaire was first prepared. This included using an open narrative at the end for the research team members to document further details or observations regarding adolescent health concerns or barriers to care. We incorporated validated questionnaire items: the Patient Health Questionnare-2 (PHQ-2) screened for symptoms of depression; the Generalized Anxiety Disorder-2 (GAD-2) screened for symptoms of anxiety; and a validated questionnaire was used to assess ART adherence [35–40]. Participants were asked if there were times that they lacked food (either none, some, or most of the time) and sources of food were queried. Study staff were directed to immediately refer participants to clinic staff if their responses indicated acute medical or psychological needs, or food insecurity. The care program liaised with ALHIV to ensure that needs were met, including by re-engaging participants that had been LTP, ensuring access to ART, and other forms of outreach. Finally, the questionnaire included information scripts regarding precautions to stay safe from COVID-19 infection, including the importance of social distancing, wearing masks, frequent hand-washing, isolating when sick, and both maintaining a supply of ART and adhering to ART. Detailed information was included that could be provided for respondents that had specific questions about COVID-19.

Study staff made three attempts to contact participants from the parent study by phone. When the adolescent could not be reached, surveys were conducted with the primary caregiver whom had consented for the adolescent’s participation in the parent study. In these cases, the caregiver was asked to respond on behalf of the adolescent about the impact of the pandemic on the adolescent. Surveys were conducted by research staff fluent in Swahili and/or Dholuo, and English, with advanced training and extensive experience working with ALHIV and their caregivers. Phone surveys were carried out from August 12 to October 19, 2020, in Eldoret; July 22 to October 30 in Kitale; and December 22, 2020, to January 7, 2021, in Kisumu.

### Ethics

All participants provided informed consent to the parent study, which included consent to provide contact information and to be contacted by the study team for additional assessments. Participants were contacted by phone to ask if they were interested in participating in this study. Verbal informed consent was obtained using standard phone consent procedures. These included procedures to confirm that the research team member was speaking to the correct person and that participants were free to talk in a private location, and if not, a plan was made for a time to call back. A verbal consent script, including information about the survey and participation, was read aloud to participants over the phone, in either English, Kiswahili, or Dholuo, and allowed for participants to ask any questions, to take time to consider participation, and/or to consider enrollment in the study at a later time. Verbal affirmation of consent to participate was required and documented by the study team. For adolescent minors, both adolescent assent and consent of their primary caregiver were required.

The study protocol was approved by the Institutional Research and Ethics Committee constituted jointly by Moi University College of Health Sciences and MTRH; the Kenya Medical Research Institute’s Scientific Ethical Review Unit; and by the Institutional Review Board at Indiana University.

### Analysis

Survey responses were analyzed through basic descriptive statistics, using STATA 14.1. Comparisons were made among adolescents who had been either retained in care or LTP at enrollment using Fisher’s exact test or Mann-Whitney test as appropriate. Analysis of PHQ-2 and GAD-2 assessments excluded caregiver respondents. Open-ended responses were categorized and quantitatively summarized when feasible, or qualitatively summarized through a simple thematic analysis.

### Rapid dissemination and response

In the initial weeks of conducting phone surveys at AMPATH sites, preliminary trends in survey responses were relayed to program leadership, and supported the rapid implementation of a dedicated philanthropic effort, which included emergency support for food distribution; cash transfers to microfinance groups and directly to PLHIV; and health system needs amid the pandemic. The final survey results were shared with leadership from the care programs.

## Results

The parent study is comprised of 378 ALHIV, including 308 who were retained in care and 70 who had been LTP at initial enrollment. Of this cohort, phone surveys were completed with 334 ALHIV or their caregivers (Table 1), including 275/308 (89.3%) participants from the retained group and 59/70 (84.3%) from the LTP group. Among all adolescents, the median age was 17.1 years (interquartile range 15.1–19.6) at the time of the survey. Females comprised 51.5%. Ninety-one percent of survey respondents were adolescents themselves; 8.7% of all respondents were primary caregivers to ALHIV in the ACE cohort. A greater proportion of surveys were completed by caregivers for ALHIV in the LTP group (27.1% vs. 4.7%, p<0.001).

**Table 1:** Participant characteristics, COVID-19 knowledge, and preparedness among adolescents living with HIV in western Kenya, with comparisons by engagement cohort at enrollment in parent study.

	Retained	LTP	Total	P-value
	N = 275 (%)	N = 59 (%)	N = 334 (%)	
**Characteristics**				
Age, median (IQR)	16.7 (14.8, 19.6)	18.6 (16.2, 20.3)	17.1 (15.1, 19.6)	*<0*.*001*
Female	135 (49.1)	37 (62.7)	172 (51.5)	0.06
Program				0.72
FACES	51 (18.5)	12 (20.3)	63 (18.9)	
AMPATH	224 (81.5)	47 (79.7)	271 (81.1)	
Clinic site				0.06
Kitale	137 (49.8)	22 (37.3)	159 (47.6)	
Lumumba	51 (18.5)	12 (20.3)	63 (18.9)	
MTRH	23 (8.4)	2 (3.4)	25 (7.5)	
Rafiki	64 (23.3)	23 (39.0)	87 (26.0)	
Calendar year of HIV care enrollment, median (IQR)	2009 (2007, 2012)	2012 (2008, 2015)	2009 (2007, 2012)	*0*.*003*
Caregiver education level				1
None	5 (3.1)	0	5 (2.7)	
Primary school (1^st^-7^th^ class)	73 (44.8)	11 (50.0)	84 (45.4)	
Secondary school (8^th^-10^th^ class/basic technician)	59 (36.2)	8 (36.4)	67 (36.2)	
Pre-University (11^th^-12^th^/medium technician/professional)	21 (12.9)	3 (13.6)	24 (13.0)	
University	5 (3.1)	0	5 (2.7)	
Caregiver respondent	13 (4.7)	16 (27.1)	29 (8.7)	*<0*.*001*
**COVID-19 Knowledge**				
Heard of COVID-19	275 (100.0)	58 (98.3)	333 (99.7)	0.177
Received official communication from HIV clinic about COVID-19	91 (33.1)	21 (36.2)	112 (33.6)	0.81
Suggestions to keep a supply of ART	55 (20.0)	17 (28.8)	72 (21.6)	0.16
Advice on how to stay healthy	86 (31.3)	18 (30.5)	104 (31.1)	1
Advice on what to do if feeling sick	49 (17.8)	10 (16.9)	59 (17.7)	1
Main sources of information about COVID-19				
Radio	194 (70.5)	40 (67.8)	234 (70.1)	0.75
TV news	227 (82.5)	42 (71.2)	269 (80.5)	0.068
Newspaper	26 (9.5)	2 (3.4)	28 (8.4)	0.19
Internet news site	15 (5.5)	4 (6.8)	19 (5.7)	0.76
Facebook	35 (12.7)	12 (20.3)	47 (14.1)	0.15
WhatsApp	21 (7.6)	11 (18.6)	32 (9.6)	*0*.*014*
Text messages	21 (7.6)	3 (5.1)	24 (7.2)	0.78
Healthcare provider/clinic	31 (11.3)	10 (16.9)	41 (12.3)	0.27
Family	142 (51.6)	21 (35.6)	163 (48.8)	*0*.*031*
Friends	117 (42.5)	18 (30.5)	135 (40.4)	0.11
Other	17 (6.2)	6 (10.2)	23 (6.9)	0.26
Symptoms independently identified for COVID-19				
Fever	189 (68.7)	46 (78.0)	235 (70.4)	0.21
Cough	222 (80.7)	50 (84.7)	272 (81.4)	0.58
Shortness of breath	137 (49.8)	34 (57.6)	171 (51.2)	0.32
Headache	128 (46.5)	21 (35.6)	149 (44.6)	0.15
Body aches	65 (23.6)	7 (11.9)	72 (21.6)	0.054
Loss of taste or smell	14 (5.1)	5 (8.5)	19 (5.7)	0.35
Vomiting	15 (5.5)	2 (3.4)	17 (5.1)	0.75
Diarrhea	5 (1.8)	0	5 (1.5)	0.59
Don’t know	17 (6.2)	1 (1.7)	18 (5.4)	0.22
Identified actions to take if sick with symptoms of COVID-19				
Isolate from others	236 (85.8)	54 (91.5)	290 (86.8)	0.29
Manage symptoms from home if able	136 (49.5)	26 (44.1)	162 (48.5)	0.48
Contact and/or visit health facility	235 (85.5)	52 (88.1)	287 (85.9)	0.68
Visit traditional healer or herbalist	12 (4.4)	0	12 (3.6)	0.14
Visit spiritual healer	9 (3.3)	0	9 (2.7)	0.37
Don’t know	9 (3.3)	0	9 (2.7)	0.37
**COVID-19 Preparedness**				
Has concerns about being able to stay healthy during pandemic	51 (18.5)	12 (20.3)	63 (18.9)	0.77
Precautions being taken to protect self from virus				
Washing hands frequently	225 (81.8)	47 (79.7)	272 (81.4)	0.71
Covering face with a mask or scarf	235 (85.5)	55 (93.2)	290 (86.8)	0.14
Avoiding touching face	50 (18.2)	5 (8.5)	55 (16.5)	0.081
Avoiding people who are sick	40 (14.5)	5 (8.5)	45 (13.5)	0.29
Avoiding visitors or social gatherings	211 (76.7)	28 (47.5)	239 (71.6)	*<0*.*001*
Avoiding public transportation	31 (11.3)	6 (10.2)	37 (11.1)	1
Not going to work site or not going to do informal work	13 (4.7)	1 (1.7)	14 (4.2)	0.48
Not going to church	14 (5.1)	0	14 (4.2)	0.14
Reasons for having to leave home				
Going to work site	50 (18.2)	14 (23.7)	64 (19.2)	0.36
Getting food our household supplies	129 (46.9)	40 (67.8)	169 (50.6)	*0*.*004*
Picking up medicine or accessing medical care	224 (81.5)	47 (79.7)	271 (81.1)	0.72
Caring for others	28 (10.2)	4 (6.8)	32 (9.6)	0.63
Going to church	101 (36.7)	18 (30.5)	119 (35.6)	0.45

A number of survey findings were similar between the two groups, and are therefore presented for the total cohort. Statistically significant differences between the groups are specified.

### COVID-19 knowledge and preparedness

Among survey respondents, nearly all (99.7%) had heard of COVID-19, with the main sources of information on COVID-19 being TV news (80.5%), radio (70.1%), family (48.8%), or friends (40.4%). Some respondents (112, 33.6%) reported having received official communication from their HIV clinic site regarding COVID-19.

Knowledge and awareness of COVID-19 symptoms included unprompted identification of symptoms such as fever (70.4%), cough (81.4%), shortness of breath (51.2%), or headache (44.6%). Knowledge surrounding actions to take if one was feeling sick with symptoms of possible COVID-19 included isolating from others (86.8%), contacting or visiting a health facility (85.9%), or managing symptoms at home if able (48.5%). Few patients were unsure of what actions one would need to take (2.7%).

Some respondents (18.9%) expressed concerns about being able to stay healthy and safe during the pandemic. Sources of greatest concern centered around fears that HIV infection may confer greater risk of illness if exposed to SARS-CoV-2. Adolescents reported needing to leave home to pick up medicine or access care (81.1%), get food or supplies (50.6%), go to church (35.6%), or go to work (19.2%), and they feared becoming infected with SARS-CoV-2 in these settings or on public transportation. Some reported lacking access to masks. Others expressed concerns about being able to eat nutritious food to stay healthy while facing significant food insecurity. All respondents reported taking measures to prevent SARS-CoV-2 infection, with most common including washing hands frequently (81.4%), covering one’s face with a mask or a covering (86.8%), avoiding visitors or social gatherings (47.5% among LTP vs. 76.7% among retained, p<0.001), and avoiding public transportation (11.1%).

### Social and economic situation

At the time phone surveys were conducted, ALHIV were staying primarily at their usual residence (66.1% among LTP, 78.2% among retained); others had gone to a rural home (20.3% among LTP, 11.3% among retained); had gone to a relative (6.8% among LTP, 4.7% among retained); or were living in a group home or institution (5.1% among LTP, 2.9% among retained; Table 2). A greater proportion of LTP adolescents had left their usual residence, though this was not statistically significant (33.9% vs. 21.8%, p = 0.189).

**Table 2:** Caregiver- and adolescent-reported social and economic impacts of the COVID-19 pandemic among adolescents living with HIV in western Kenya, with comparisons by engagement cohort at enrollment in parent study.

	Retained	LTP	Total	P-value
	N = 275 (%)	N = 59 (%)	N = 334 (%)	
**Social and Economic Situation**				
Living situation during the pandemic				0.19
Usual residence	215 (78.2)	39 (66.1)	254 (76.0)	
Rural home	31 (11.3)	12 (20.3)	43 (12.9)	
Relative’s home	13 (4.7)	4 (6.8)	17 (5.1)	
Friend’s home	1 (0.4)	0	1 (0.3)	
Work site	1 (0.4)	0	1 (0.3)	
School	4 (1.5)	0	4 (1.2)	
Institution / group home	8 (2.9)	3 (5.1)	11 (3.3)	
Street-connected	0	1 (1.7)	1 (0.30)	
Enrolled in school	234 (85.1)	37 (62.7)	271 (81.1)	*<0*.*001*
Impact on schooling				*0*.*017*
No longer doing school work	100 (36.4)	27 (45.8)	127 (38.0)	
No longer going to classes in person but still completing school work	128 (46.5)	17 (28.8)	145 (43.4)	
No impact	47 (17.1)	14 (23.7)	61 (18.3)	
History of working or earning income prior to the pandemic	26 (9.5)	9 (15.3)	35 (10.5)	0.24
Formal or informal sector, N = 34				0.064
Formal sector	0	2 (22.2)	2 (5.9)	
Informal sector	25 (100)	7 (77.8)	32 (94.1)	
Public-facing work (e.g. *matatu* driver, waiter/waitress, shopkeeper), N = 35	18 (69.2)	9 (100.0)	27 (77.1)	0.081
Loss of adolescent’s job/income, N = 35				0.64
Lost formal job	4 (15.4)	1 (11.1)	5 (14.3)	
Lost other income	14 (53.8)	7 (77.8)	21 (60.0)	
No loss of job or income	8 (30.8)	1 (11.1)	9 (25.7)	
Others at home relying on adolescent’s income, N = 35	21 (80.8)	6 (66.7)	27 (77.1)	0.40
Others at home (whom the adolescent relies on) have lost job or lost income	98 (35.6)	22 (37.3)	120 (35.9)	0.93
Food insecurity during the pandemic				0.31
Lacking food some of the time	97 (35.3)	24 (40.7)	121 (36.2)	
Lacking food most of the time	10 (3.6)	4 (6.8)	14 (4.2)	
There has been enough food	168 (61.1)	31 (52.5)	199 (59.6)	
Sources of food during the pandemic				
Stores / markets	242 (88.0)	43 (72.9)	285 (85.3)	*0*.*007*
Own farm	145 (52.7)	34 (57.6)	179 (53.6)	0.57
Relative or friend’s farm	38 (13.8)	8 (13.6)	46 (13.8)	1
Needed to travel from town to rural area because of pandemic	46 (16.7)	4 (6.8)	50 (15.0)	0.13

A greater proportion of LTP adolescents were not enrolled in school (37.3% among LTP vs. 14.9% among retained, p<0.001), or were no longer doing school work (45.8% among LTP vs. 36.4% among retained, p = 0.017).

Prior to the pandemic, 35 (10.5%) of adolescents were working or earning income, primarily in the informal sector (94.1%) and mostly in public-facing jobs (77.1%). Of these, 26 (74.3%) adolescents lost their job or income during the pandemic. Over a third of adolescents (120, 35.9%) had relied on someone else at home who lost a job or lost income during the pandemic.

In total, 135 adolescents (40.4%) did not have enough food either some (121, 36.2%) or most (14, 4.2%) of the time. Adolescents’ primary sources of food during the pandemic were stores or markets (72.9% among LTP vs. 88.0% among retained, p = 0.007), their own farms (53.6%), a relative’s or friend’s farm (13.8%), or other sources (2.4%), which included food distributions from church groups, AMPATH, or other organizations.

In open-ended responses regarding major life changes during the pandemic, respondents emphasized challenges from being out of school, missing socialization with peers, as well as lacking money for food or basic needs, and a lack of work for themselves or their families.

### Access to antiretroviral therapy

Regarding their ART medications, 99.4% of adolescents currently had ART with them or where they have been sheltering (Table 3). Two adolescents in the LTP group did not have ART; they had experienced 90 days and 330 days without ART, respectively. Of those with ART, 4.2% reported having less than a one-week supply. Seventeen percent of adolescents had concerns about running out of ART during the pandemic, and 5.1% reported skipping medication doses due to concerns about running out of ART medications. More adolescents in the LTP group (6.8%) than in the retained group (0.7%, p = 0.010) reported greater difficulties refilling medications during the pandemic. Challenges included lacking transportation (n = 4); curfew or travel restrictions (1); fear of traveling (1); restrictions on entering hospital site (1); and medication stock-out (1).

**Table 3:** Caregiver- and adolescent-reported ART access and adherence among adolescents living with HIV during the COVID-19 pandemic in western Kenya, with comparisons by engagement cohort at enrollment in parent study.

	Retained	LTP	Total	P-value
	N = 275 (%)	N = 59 (%)	N = 334 (%)	
**Access to ART**				
Currently have ART with them / at home	275 (100.0)	57 (96.6)	332 (99.4)	*0*.*031*
Among those with ART, supply remaining, N = 332				0.38
≤1 week	9 (3.3)	5 (8.8)	14 (4.2)	
>1 week and ≤30 days	112 (40.7)	27 (47.4)	139 (41.9)	
>30 days and ≤60 days	84 (30.5)	16 (28.1)	100 (30.1)	
>60 days and ≤90 days	48 (17.5)	8 (14.0)	56 (16.9)	
>90 days	13 (4.7)	1 (1.8)	14 (4.2)	
Don’t know	8 (2.9)	0	8 (2.4)	
Has concerns about running out of ART	46 (16.7)	10 (16.9)	56 (16.8)	0.44
Has skipped doses during pandemic due to concerns of running out	15 (5.5)	2 (3.4)	17 (5.1)	0.21
Has had greater difficulties filling medications during pandemic	2 (0.7)	4 (6.8)	6 (1.8)	*0*.*010*
Transportation too expensive or not available	1 (0.4)	3 (5.1)	4 (1.2)	*0*.*018*
Curfew, restrictions on travel, or police crackdowns	0	1 (1.7)	1 (0.3)	0.18
Fear of traveling	0	2 (3.4)	2 (0.6)	*0*.*031*
Other	1 (0.4)	1 (1.7)	2 (0.6)	0.32
**ART Adherence**				
Do you have problems taking your ART?				0.18
Yes	10 (3.6)	3 (5.1)	13 (3.9)	
No	265 (96.4)	55 (93.2)	320 (95.8)	
N/A, not on treatment	0	1 (1.7)	1 (0.3)	
Have you forgotten to take your ART within the last 3 days				*0*.*045*
Yes	17 (6.2)	4 (6.9)	21 (6.3)	
No	258 (93.8)	52 (89.7)	310 (93.1)	
Don’t know	0	1 (1.7)	1 (0.3)	
Prefer not to answer	0	1 (1.7)	1 (0.3)	
Do you have problems taking your ART around others?				0.47
Yes	51 (18.5)	14 (24.1)	65 (19.5)	
No	223 (81.1)	44 (75.9)	267 (80.2)	
Prefer not to answer	1 (0.4)	0	1 (0.3)	
Have you taken a dose more than 1 hour late in the past 7 days?				*0*.*027*
Yes	57 (20.7)	8 (13.8)	65 (19.5)	
No	218 (79.3)	48 (82.8)	266 (79.9)	
Don’t know	0	1 (1.7)	1 (0.3)	
Prefer not to answer	0	1 (1.7)	1 (0.3)	
Are there times when you are supposed to take the medicine but you don’t have them with you?				0.56
Yes	17 (6.2)	5 (8.6)	22 (6.6)	
No	258 (93.8)	53 (91.4)	311 (93.4)	
Have you missed at least one dose in the past 7 days				0.099
Yes	19 (6.9)	2 (3.4)	21 (6.3)	
No	255 (92.7)	54 (93.1)	309 (92.8)	
Don’t know	1 (0.4)	1 (1.7)	2 (0.6)	
Prefer not to answer	0	1 (1.7)	1 (0.3)	
Have made changes in taking ART as a result of pandemic	28 (10.2)	8 (14.3)	36 (10.9)	0.35

### Antiretroviral adherence

Thirteen (3.9%) adolescents reported problems taking ART. Open-ended responses regarding challenges taking ART included forgetting doses (n = 3), not taking ART while not having food (n = 3), avoiding taking ART around others (n = 2); some described these challenges as specifically due to pandemic-related changes in schedules or in living situations. Forgetting to take ART within the last 3 days was more frequent among LTP ALHIV (6.9% among LTP vs. 6.2% among retained, p = 0.045). Taking a dose >1 hour late in the last 7 days was more frequent among retained ALHIV (13.8% among LTP vs. 20.7% among retained, p = 0.027). Twenty-one (6.3%) adolescents missed at least 1 dose in the past 7 days. Twenty-two (6.6%) adolescents experienced times that they were supposed to take their ART, but did not have it with them. Many adolescents (36, 10.9%) had made changes in the way they take ART during the pandemic. These included changes to their medication schedule (n = 22), regimen (n = 6), lacking trimethoprim-sulfamethoxazole (n = 3), as well as improved adherence (n = 2), and restarting ART (n = 2).

### Health status, health-seeking behavior, and healthcare access

Among respondents, 44 (13.2%) adolescents had felt ill at some point in the preceding 2 months. Of these, 36 (80%) sought care at the HIV clinic (n = 19), a hospital (n = 10), or a pharmacist (n = 7) (Table 4). Five adolescents were continuing to feel unwell at the time of the survey. Illness symptoms at any point during the prior 2 months included: fever (9.6%), cough (9.9%), shortness of breath (3.0%), fatigue (4.8%), body aches (7.2%), congestion (12.0%), sore throat (3.6%), vomiting (2.1%), or diarrhea (4.2%). One adolescent had been hospitalized during the previous 2 months, for severe malaria.

**Table 4:** Caregiver- and adolescent-reported health status and health-seeking behavior among adolescents living with HIV during the COVID-19 pandemic in western Kenya, with comparisons by engagement cohort at enrollment in parent study.

	Retained	LTP	Total	P-value
	N = 275 (%)	N = 59 (%)	N = 334 (%)	
**Health Status and Health-Seeking Behavior**				
Have felt ill during the past two months	35 (12.7)	9 (15.3)	44 (13.2)	0.67
Have sought care, N = 45	29 (80.6)	7 (77.8)	36 (80.0)	1
Where sought care				
Clinic	17 (6.2)	2 (3.4)	19 (5.7)	0.55
Hospital	6 (2.2)	4 (6.8)	10 (3.0)	*0*.*08*
Pharmacist	6 (2.2)	1 (1.7)	7 (2.1)	1
Continuing to feel unwell, N = 36	4 (13.8)	1 (14.3)	5 (13.9)	1
Having greater difficulties than usual in getting medical care during the pandemic	17 (6.2)	1 (1.7)	18 (5.4)	0.081
What difficulties have you had getting medical care?				
Clinic or facility not open / not functioning / stock-outs	1 (0.4)	0	1 (0.3)	1
Transportation too expensive or not available	11 (4.0)	0	11 (3.3)	0.22
Curfew, restrictions on travel, or police crackdowns	1 (0.4)	0	1 (0.3)	1
Fear of traveling	3 (1.1)	0	3 (0.9)	1
Lack of funds for medications	3 (1.1)	1 (1.7)	4 (1.2)	0.54
Symptoms experienced during the past 2 months				
Fever	26 (9.5)	6 (10.2)	32 (9.6)	0.81
Cough	25 (9.1)	8 (13.6)	33 (9.9)	0.34
Shortness of breath	7 (2.5)	3 (5.1)	10 (3.0)	0.39
Fatigue	12 (4.4)	4 (6.8)	16 (4.8)	0.50
Body aches	20 (7.3)	4 (6.8)	24 (7.2)	1
Congestion	34 (12.4)	6 (10.2)	40 (12.0)	0.83
Sore throat	10 (3.6)	2 (3.4)	12 (3.6)	1
Vomiting	5 (1.8)	2 (3.4)	7 (2.1)	0.36
Diarrhea	14 (5.1)	0	14 (4.2)	0.14
Other	11 (4.0)	3 (5.1)	14 (4.2)	0.72
No symptoms	193 (70.2)	44 (74.6)	237 (71.0)	0.53
Need for hospitalization during the past 2 months	1 (0.4)	0	1 (0.3)	1
Had a laboratory test for COVID-19, N = 333	7 (2.6)	1 (1.7)	8 (2.4)	1
Had a diagnosis of COVID-19 (either by testing or clinical suspicion)	0	0	0	
Household members or other close contacts have been ill	7 (2.5)	2 (3.4)	9 (2.7)	0.66
Household members or other close contacts diagnosed with COVID-19				1
Yes, based on positive test	3 (1.1)	0	3 (0.9)	
Yes, unsure how determined	1 (0.4)	0	1 (0.3)	
Prefer not to answer	1 (0.4)	0	1 (0.3)	
No	270 (98.2)	59 (100.0)	329 (98.5)	

Eight adolescents (2.4%) had been tested for COVID-19 at any time. No adolescents had been told by a medical provider that they had COVID-19. Nine adolescents (2.7%) had reportedly had close contacts who had been ill, including four who reported contacts with individuals diagnosed with COVID-19.

Eighteen adolescents (5.4%) experienced greater difficulties than usual in accessing medical care. These included challenges due to limited access to transportation (11), lack of money for medications (4), fear of traveling (3), travel restrictions (1), or clinic closures or stock-outs (1).

### Mental health

Adolescent scores on the PHQ-2 were categorized as 0–2 (94.4%) vs. 3 or higher (5.6%), with those 3 or higher meeting the threshold for further evaluations for possible depression (Table 5). Similarly, scores on the GAD-2 ranged from 0–2 (94.8%) to 3 or higher (5.2%), with those 3 or higher meeting the threshold for further evaluations for possible anxiety.

**Table 5:** Responses to mental health screening questionnaires among adolescents living with HIV in western Kenya during the COVID-19 pandemic, with comparisons by engagement cohort at enrollment in parent study.

	Retained	LTP	Total	P-value
	N = 262 (%)	N = 43 (%)	N = 305 (%)	
**Mental Health**				
**PHQ-2**				
Little interest or pleasure in doing things in the past 2 weeks				0.45
Not at all (0)	230 (87.8)	36 (83.7)	266 (87.2)	
Several days (1)	21 (8.0)	5 (11.6)	26 (8.5)	
More than half of the days (2)	6 (2.3)	2 (4.7)	8 (2.6)	
Nearly every day (3)	5 (1.9)	0	5 (1.6)	
Feeling down, depressed, or hopeless in the past 2 weeks				0.59
Not at all (0)	222 (84.7)	37 (86.0)	259 (84.9)	
Several days (1)	27 (10.3)	6 (14.0)	33 (10.8)	
More than half of the days (2)	10 (3.8)	0	10 (3.3)	
Nearly every day (3)	3 (1.1)	0	3 (1.0)	
PHQ-2 Scores				1
0–2	247 (94.3)	41 (95.3)	288 (94.4)	
3–6	15 (5.7)	2 (4.7)	17 (5.6)	
**GAD-2**				
Feeling nervous, anxious, or on edge in the past 2 weeks				0.59
Not at all (0)	225 (85.9)	36 (83.7)	261 (85.6)	
Several days (1)	20 (7.6)	6 (14.0)	26 (8.5)	
More than half of the days (2)	11 (4.2)	1 (2.3)	12 (3.9)	
Nearly every day (3)	5 (1.9)	0	5 (1.6)	
Prefer not to answer	1 (0.4)	0	1 (0.3)	
Not being able to stop or control worrying in the past 2 weeks				0.76
Not at all (0)	226 (86.3)	39 (90.7)	265 (86.9)	
Several days (1)	22 (8.4)	4 (9.3)	26 (8.5)	
More than half of the days (2)	8 (3.1)	0	8 (2.6)	
Nearly every day (3)	6 (2.3)	0	6 (2.0)	
GAD-2 Scores				0.14
0–2	246 (93.9)	43 (100.0)	289 (94.8)	
3–6	16 (6.1)	0	16 (5.2)	

### Overall impacts

On completion of phone surveys, the research team members were prompted to document details or observations regarding adolescent health concerns or barriers to care. These descriptions centered primarily on food insecurity, challenges meeting basic needs, and lack of affordable transportation. Some described extreme hardships which impeded care; these included experiences related to navigating pregnancy and HIV care during the pandemic, affording basic needs, leaving home, or being relocated from an orphanage that had closed. Several described adolescents’ challenges taking ART, due to lacking food, difficulty finding privacy to take ART, or worsened mental health; some adolescents had reportedly discontinued ART. Care was disrupted when adolescents were unable to afford transportation or medications, or feared coming to clinic due to COVID-19. Adolescents had also reported ways in which their clinic had supported continuity for their treatment despite significant obstacles posed by the pandemic.

## Discussion

In this cross-sectional survey of ALHIV currently or previously enrolled in HIV care, we observed multiple large-scale effects of the COVID-19 pandemic on schooling, income, and financial and food security for ALHIV and their households. More than a third of participants were no longer doing any school work. Of those who had been working, most had lost a job or lost income due to the pandemic. More than a third had experienced the loss of income for a family member on whom they relied, and more than 40% experienced food insecurity. Despite widescale economic, social, and healthcare challenges, few adolescents reported lacking ART, and we observed relatively low proportions of reported ART adherence challenges. Amid significant societal disruptions in western Kenya, and globally, during an unprecedented pandemic, it is and will remain essential to address the economic and social dimensions of the pandemic, particularly as they affect ALHIV, young people, and other vulnerable groups. Identifying areas and opportunities to further strengthen health systems to sustain HIV care engagement and access is of critical ongoing importance.

Surveyed ALHIV who were LTP at enrollment in the parent study had notable differences from the retained group, including being older, with a greater proportion of females, enrolled at the Rafiki Center, and initially enrolled in HIV care in later calendar years. The Rafiki Center serves a long-standing cohort of ALHIV, and includes a clinic population with particularly complex medical and social vulnerabilities, including orphaned and street-connected youth. Later age at HIV care enrollment may signal greater medical vulnerability and barriers to engagement, and both females and older adolescents have been observed to have risks for disengagement [10, 41, 42]. In this study we examined how these groups with differing care engagement at enrollment in the parent study experienced a range of impacts of the COVID-19 pandemic.

By some measures, greater social, economic, and health challenges associated with the pandemic were observed among ALHIV LTP at enrollment into the parent study compared to those engaged in HIV care. These included that among LTP ALHIV, a greater proportion had stopped schooling, or had left their usual residence due to the pandemic. Further, a larger proportion of LTP ALHIV experienced greater difficulties filling ART medications during the pandemic, or had forgotten to take ART in the past 3 days. Such differences might relate to underlying social vulnerabilities that drive disengagement from HIV care and which may exacerbate the impact of societal disruptions during the COVID-19 pandemic [8, 9, 11, 34]. At the same time, large proportions of *all* ALHIV in this study were experiencing financial or food insecurity and/or interruptions to school or work, reflecting widescale challenges affecting adolescents and their households. Further findings include that LTP ALHIV were less likely to avoid visitors or social gatherings. It is unclear if this finding may possibly relate to any underlying differences in social factors, such that they were less able to avoid visitors or social gatherings, or different sources of information regarding COVID-19, with the LTP group reporting higher proportions of e.g. WhatsApp or Facebook as main sources of information, and lower use of TV news or newspaper sources as compared to the retained group. In either case, further work may be needed for infection prevention messaging and interventions to mitigate risk of COVID-19 exposure to vulnerable ALHIV.

Very few studies have been conducted regarding the impacts of the COVID-19 pandemic among ALHIV or in sub-Saharan Africa, despite the particular vulnerability of this population, most of whom are living in eastern and southern Africa [43, 44]. One study of ALHIV in Kenya also found significant social and economic impacts [45]. The present study included a cohort of ALHIV with recent care disengagement, a group for whom the pandemic effects appeared to be more significant, as well as some more detailed assessments than in the previous study. Both studies strongly emphasize the urgent, acute economic and social impacts of the pandemic experienced by ALHIV in this setting.

As phone surveys were initiated in this and other PLHIV cohorts in the IeDEA East Africa Region, emergent needs of this population were immediately apparent. The majority of participants in this study were given referrals to either social work, clinical teams, or partnering community organizations, to address immediate needs including those food security, access to ART, costs of transportation to clinic, needs for pregnant adolescents, or other acute concerns regarding access to healthcare or education. Further, initial survey findings were used to support an emergency philanthropic effort at AMPATH, which included food distribution and economic support to PLHIV, as well as funding for health system capacity for managing COVID-19 patients. The health system was exceedingly strained by several factors, including patient care needs, limited availability of PPE, and by the deaths of several clinicians and staff. For ALHIV, a previously adolescent-dedicated clinic was re-purposed as a COVID-19 isolation ward, necessitating the temporary relocation of adolescent HIV services to a main clinic site which was not solely for adolescent care. This change, coupled with some turnover or sharing of clinic staff, as well as the loss of in-person adolescent support groups, presented multiple challenges to HIV care for adolescents. Severe stressors on health systems in the context of the pandemic limit their capacity to respond to the needs of PLHIV. We observed ways in which ALHIV may be particularly vulnerable amid widescale challenges to the health system.

Some findings from this study may point to areas of resilience in both the health system and among ALHIV to sustain HIV care engagement during a massively disruptive period. Among the cohort that was reached for this study, only two adolescents did not have ART with them; and 14 (4.2%) had less than one week of medication remaining. Both HIV care teams and the research team undertook intensive efforts from the start of the pandemic to ensure continued ART access among ALHIV in this group. These included outreach phone calls; provision of multi-month refills, ensuring refills, labs, or transfer of care at local facilities when needed; and working to ensure access to ART and to basic needs. At AMPATH, youth peer mentors had critical roles in ensuring access to ART and supporting retention among adolescents during this period, and have served to promote resilience in HIV clinical care during this crisis. The sustained ART access in this group may be compared with a study which demonstrated sustained ART provision for people treated for HIV in primary clinics in South Africa during the strict initial lockdown period [46].

Meanwhile, we observed that a minority of participants reported receiving official communication from their HIV clinic about COVID-19. We note that it is possible that some participants had been contacted but may not have considered such contacts as formal communications or as explicitly regarding COVID-19. It is also possible that the clinic had attempted to contact but was unable to reach others. There may be opportunities for further enhancing health system communications, including 2-way communications to identify and respond to patient needs, particularly for potentially vulnerable populations including people living with HIV. Enhanced communication may be needed both to disseminate health information, such as when symptoms may be managed at home, if appropriate, and to be responsive to emerging needs, during a crisis period such as this.

Regarding adherence, a slightly lower proportion of adolescents reported ART adherence challenges than in previous studies. Overall proportions of taking a dose >1 hour late in the last 7 days, missing at least 1 dose in the past 7 days, and forgetting to take ART within the last 3 days were 19.5%, 6.3%, and 6.3%, respectively. Studies of adolescent- or caregiver-reported adolescent adherence to ART in this setting have previously noted slightly higher proportions of taking ART >1 hour late in the last 7 days (26–29%); missing at least 1 dose in the past 7 days (16–18%); and forgetting to take ART within the last 3 days (8%) [47–49]. It is possible that respondents were less likely to report adherence challenges in phone surveys than in in-person studies. Nevertheless, this finding suggests that ALHIV in this group did not experience a higher degree of ART adherence challenges than is typical, and may have even had slightly better adherence as a group during this period. While some adolescents noted barriers to adherence, such as being around new household members to whom they had not disclosed their status, others described in open-ended items experiencing improved adherence or re-starting ART during this period. Described facilitators to adherence included motivation to stay healthy amid the pandemic, and not being in school (where anticipated stigma from peers may deter adherence). Several described multiple efforts taken by the clinic to sustain ART access and engage adolescents around any barriers to adherence or care, all of which may have helped promote adherence. Clinic efforts included frequent communication and support by phone and use of peer mentors in additional roles to support adolescent engagement. Further research is needed to understand impacts on ART adherence over the time course of the pandemic.

During a prior humanitarian crisis of the 2007–2008 post-election conflict in Kenya, impacts were observed on pediatric ART adherence and loss to follow-up [33]. It was noted, however, that perhaps the effects were not as great as would have been expected given the scope of conflict and displacement in western Kenya [33]. Then, as in now, it became apparent that while children and adolescents with HIV are vulnerable to large-scale crises disrupting care engagement, the resilience and responsiveness of the health program in these emergencies critically enabled sustained pediatric HIV care [30, 33]. Proactively building on and strengthening key supports such as systems for tracking and following up patients, provision of multidisciplinary support including peer support, and optimizing use of mobile phones for care support and coordination can further support care continuity during future periods of social crises or disruptions.

On mental health screens, 5–6% of adolescent respondents met thresholds to further evaluate for possible depression or anxiety, while greater numbers reported any degree of health symptoms on individual PHQ-2 and GAD-2 items (13.1%-15.1%). A separate study conducted with ALHIV in western Kenya during the COVID-19 pandemic found that 5–6% of ALHIV had PHQ-9 scores consistent with mild to severe depression; compared with 21% of young adults (20–24 years old) [45]. Limited data are available regarding the prevalence of mental health disorders among ALHIV in African countries [22]. A recent pilot study in this population reported depression 3.3% and anxiety 6.7% on baseline assessments [47]. Larger studies among ALHIV in Kenya and other countries in the region have found higher rates of depression (17.8%-25%) and anxiety (32.3%) [50–53]. Challenges exist measuring mental health across global settings, and there is a need to rigorously evaluate and validate mental health instruments for use with this population [22]. As we noted for self-reported adherence, while it is possible that use of these instruments in phone surveys may have limited sensitivity to detect mental health symptoms, given that the measured prevalence of symptoms was lower than might have been expected in this population and in a challenging time period, we must also consider the resilience of this group of ALHIV. Further study may clarify factors promoting mental health among ALHIV over this period, and should include longitudinal assessments of mental health.

As was the case before the COVID-19 pandemic, strategies are needed to support engagement in care and positive mental health among PLHIV across settings. Important areas of intervention include provision of social, economic, nutritional, and mental health support, upholding human rights, and addressing health disparities [3, 14, 54]. There may be important roles for mobile strategies for provision of HIV or mental health care, as well as for decentralized HIV care and multi-month ART refills [14, 15, 17]. On a practical level, this includes developing strategies for clinics to identify and respond to emerging needs of PLHIV, to adapt clinic services to virtual/phone modalities or longer follow-up times, and to facilitate in-person care when needed [18, 55, 56]. As adolescents have unique vulnerabilities, ensuring continuation of adolescent-dedicated services, including social and peer support, and adapting these services as feasible during the pandemic, is critical [18]. More broadly, children and young people living in sub-Saharan Africa may experience an array of social and health impacts from the pandemic, including those living with HIV, TB, or other health conditions, and approaches are needed to mitigate the broad impacts of the pandemic for young people, including on their education and healthcare access [3, 5].

Our study has several limitations. First, responses were self-reported and therefore prone to social desirability and other reporting biases. It is therefore possible that some impacts of the pandemic may be under-reported, including those related to ART adherence or mental health symptoms. Second, while we reached 88.4% of participants in the parent study, the response rate was lower among those recently LTP (84%), and some surveys were completed by primary caregivers which may be subject to measurement error. It is possible, if not likely, that social and economic impacts, as well as impacts on health access and ART adherence may be greater among the LTP ALHIV or caregivers who could not be reached. Nevertheless, the inclusion of a cohort of adolescents with histories of recent care disengagement is valuable, and we noted some important differences in effects of the pandemic affecting this group. We note that over the time course that surveys were conducted, some factors changed related to the pandemic and the healthcare response. For example, in later surveys, some respondents reported receiving food through a food distribution effort that was prompted by initial surveys. A further limitation is that in this study we do not have comparable data with other groups, such as PLHIV, or adolescents who do not have HIV infection, and therefore we cannot draw conclusions about the psychosocial impacts of the pandemic on ALHIV as compared to other populations in Kenya. Finally, we note that the comparisons in this study by engagement history are not explanatory, but rather describe responses according to baseline engagement groups; and as unadjusted analyses they do not control for confounding factors.

In this study we were able to examine ALHIV cohorts according to recent histories of care disengagement, as determined at their enrollment in an ongoing prospective study. This study does not reflect their current engagement in care, which might have changed during the pandemic. We observed that most ALHIV in this study, including those in the LTP cohort, had access to ART, which may reflect either recent care re-engagement and/or the efforts of the care program and the study team to ensure ART access. With the onset of the COVID-19 pandemic, HIV programs in this setting worked to ensure that all PLHIV had access to ART, including providing multi-month refills and refills at local dispensaries, and deferring clinic visits when appropriate. The study team also liaised with all ALHIV in this cohort to understand acute needs and ensure access to ART. Engagement in HIV care is not a static outcome; there is “churn” over time for individuals into and out of care [57–59]. It is valuable to understand how previous disengagement relates to health outcomes over time, and this study provides a “snapshot” of ALHIV health during a crisis period. It will be important to assess the trajectories of adolescents over the course of the pandemic, with regard to their re-engagement or disengagement from care over time, given the significant economic, healthcare, and social challenges wrought by the pandemic.

## Conclusions

The COVID-19 pandemic has had devastating effects on education, and financial and food security for ALHIV and their households in western Kenya. ALHIV who experienced challenges with care retention before the pandemic may be especially vulnerable. Given the extent of educational, financial, and food security impacts of the pandemic, there is a risk for potential lasting effects of the pandemic which compromise adolescent health and trajectories into stable adulthoods. In this context, HIV treatment programs should consider approaches to support immediate and ongoing needs for patients and families, such as through nutritional or financial supports. At the same time, we observed sustained ART access, and lower self-reported adherence challenges or mental health symptoms than might have been anticipated, potentially signaling resilience and strengths of the ALHIV in this study and their care programs. It will be important to continue to assess these effects of the pandemic over time for ALHIV and to develop proactive supports for this vulnerable group during this crisis.
